# Variation in and risk factors for paediatric inpatient all-cause mortality in a low income setting: data from an emerging clinical information network

**DOI:** 10.1186/s12887-017-0850-8

**Published:** 2017-04-05

**Authors:** David Gathara, Lucas Malla, Philip Ayieko, Stella Karuri, Rachel Nyamai, Grace Irimu, Michael Boele van Hensbroek, Elizabeth Allen, Mike English, the Clinical Information Network, David Githanga, Fred Were, Barnabas Kigen, Samuel NgarNgar, Nick Aduro, Rachel Inginia, Beatrice Mutai, Grace Ochieng, Lydia Thuranira, Francis Kanyingi, Margaret Kuria, Sam Otido, Kigondu Rutha, Peris Njiiri, Martin Chabi, Charles Nzioki, Joan Ondere, Caren Emadau, Cecelia Mutiso, Naomi Muinga, Michael Bitok, Timothy Tuti, Boniface Makone, Wycliffe Nyachiro, George Mbevi, Thomas Julius, Susan Gachau, Morris Ogero

**Affiliations:** 1 Department of Public Health Research, KEMRI Wellcome Trust Research Programme, P.O. Box 43640 00100, Nairobi, Kenya; 2 Nuffield Department of Medicine, University of Oxford, Oxford, OX3 7BN UK; 3 Division of Maternal, Newborn, Child and Adolescent Health, Ministry of Health, Nairobi, Kenya; 4 Department of Paediatrics and Child Health, University of Nairobi, Nairobi, 19676-00202 Kenya; 5 Department of Global Health, Academic Medical Centre, University of Amsterdam, Amsterdam, 22700 1100 DE The Netherlands; 6 Department of Medical Statistics, London School of Hygiene and Tropical Medicine, London, WC1E 7HT UK

**Keywords:** Mortality, Quality of care, Paediatrics, Hospital, Variability, Clinical risk factors

## Abstract

**Background:**

Hospital mortality data can inform planning for health interventions and may help optimize resource allocation if they are reliable and appropriately interpreted. However such data are often not available in low income countries including Kenya.

**Methods:**

Data from the Clinical Information Network covering 12 county hospitals’ paediatric admissions aged 2–59 months for the periods September 2013 to March 2015 were used to describe mortality across differing contexts and to explore whether simple clinical characteristics used to classify severity of illness in common treatment guidelines are consistently associated with inpatient mortality. Regression models accounting for hospital identity and malaria prevalence (low or high) were used. Multiple imputation for missing data was based on a missing at random assumption with sensitivity analyses based on pattern mixture missing not at random assumptions.

**Results:**

The overall cluster adjusted crude mortality rate across hospitals was 6 · 2% with an almost 5 fold variation across sites (95% CI 4 · 9 to 7 · 8; range 2 · 1% - 11 · 0%). Hospital identity was significantly associated with mortality. Clinical features included in guidelines for common diseases to assess severity of illness were consistently associated with mortality in multivariable analyses (AROC =0 · 86).

**Conclusion:**

All-cause mortality is highly variable across hospitals and associated with clinical risk factors identified in disease specific guidelines. A panel of these clinical features may provide a basic common data framework as part of improved health information systems to support evaluations of quality and outcomes of care at scale and inform health system strengthening efforts.

## Background

It is important for a health system to have an accsurate picture of overall (crude) and cause-specific hospital mortality. Although using mortality or risk adjusted mortality as an indicator of quality of care is contested because it is hard to adjust for case-mix or the severity of illness on arrival (case-severity) [1–4] the presence of 3.variation in mortality may point to possible inequalities in population health, access or resource provision that can be addressed. Yet, little attention has been paid to understanding and exploring hospital mortality and its variability in African settings, perhaps because routinely reported data are often of poor quality [5].

In this report our aim is to contribute to efforts to understand health system performance and describe mortality and its variability. We also explore whether simple clinical characteristics used to classify severity of common childhood illness are consistently associated with inpatient mortality. Demonstrating the latter provides a rationale for reinforcing their widespread clinical use and for considering them as components of a common data framework for paediatric admissions. A common data framework (potentially included in emerging electronic record systems) could improve our ability to characterise hospitals by their case-mix and case severity and inform health system strengthening efforts in support of universal access to quality health care.

## Methods

### Study setting

In 2014, Kenya had a gross domestic product of 1246 US dollars per capita after rebasing [6] and the under-five mortality was 58.3 per 1000 live births according to 2013 estimates [7, 8]. It has good immunization coverage of 90% for the 3rd dose of the pentavalent vaccine (introduced in 2002 and containing Diphtheria, Pertussis, Tetanus, Hepatitis B and *Haemophilus influenzae* type B antigens) and 85% for the 10-valent pneumococcal conjugate vaccine (PCV 10, introduced in 2011) [7].

**Fig. 1:**
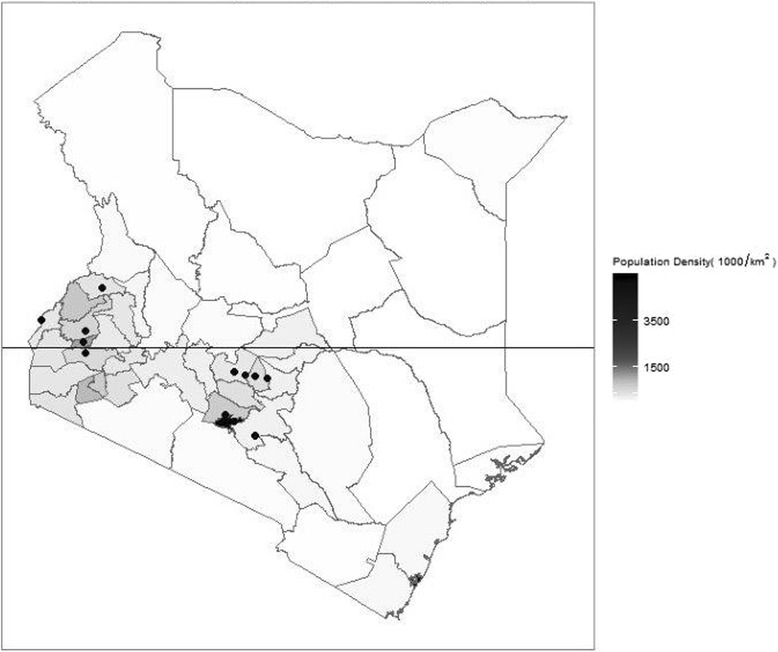
Geographic location of hospitals. Black dots represent hospitals in the clinical information network and included in the analysis while the black lines represent county boundaries. Hospitals are clustered in the central and western regions consistent with where the majority of the Kenyan population lives

The study takes advantage of a recently established clinical information network (CIN) comprised of 13 county referral hospitals. However, one county hospital was excluded from the analyses presented because of persistent problems with data collection. In total 12 facilities (11 counties) are therefore included in this analysis. The selection and geographic location of hospitals is presented in detail in panel 1, Table 1 and Fig. 1. In brief, CIN is a partnership between researchers, the Ministry of Health and paediatricians and is a pragmatic research database collecting patient level data from all paediatric admissions with aims at improving use of information in policy and practice.

**Table 1:** Characteristics of hospitals in the clinical information network

Hospital	Bed capacity	Duration of data collection in months	Cases available for analysis –minimum dataset	Cases available for analysis –full dataset	Diarrhoea admissions	Pneumonia admissions	Malaria admissions	PMTCT HIV prevalence	Percentage living in poverty in the county
A	67	18	4757	2081	1659 (34·9)	2351 (49·4)	108 (2·3)	6.7	26
B	35	18	1853	1685	446 (24·1)	1029 (55·5)	65 (3·5)	6.8	56
C	41	18	3517	1989	1063 (30·2)	1650 (46·9)	317 (9·0)	9.7	21
D	42	18	2445	2217	747 (30·6)	1420 (58·1)	210 (8·6)	9.7	21
E	29	13	1982	1774	436 (22·0)	1057 (53·3)	138 (7·0)	2.8	25
F	63	13	2440	2215	663 (27·2)	1379 (56·5)	252 (10·3)	2.8	41
G	32	13	1881	1726	391 (20·8)	886 (47·1)	10 (0·5)	5.5	31
H	29	13	2146	1767	531 (24·7)	548 (25·5)	1238 (57·7)	20.5	45
I	35	17	4175	3812	1106 (26·5)	1224 (29·3)	3640 (87·2)	4.5	51
J	21	17	2209	1729	267 (12·1)	504 (22·8)	1416 (64·1)	11.8	40
K	32	17	3066	2454	474 (15·5)	867 (28·3)	2020 (65·9)	13.9	65
L	38	17	3270	2875	967 (29·6)	1433 (43·8)	1993 (60·9)	9.3	49

Hospital workload, epidemiological diversity, catchment population poverty index and data available for the analysis

***Panel 1: Selection of the Clinical Information Network sites and Case Sampling***

Kenya devolved health care provision to 47 county administrations in 2013 with the national Ministry of Health retaining responsibility for monitoring and evaluation amongst other areas. The clinical information network (CIN) was designed as a partnership between researchers, the Ministry of Health and paediatricians and is a pragmatic research database collecting patient level data from all paediatric admissions with aims at improving use of information in policy and practice. Twelve counties were first identified purposefully with the Ministry of Health to ensure the feasibility of the project while representing two main groupings based on the prevalence of malaria as an admission clinical diagnosis: high (>50%) and low (<20%). Within counties tertiary level facilities were excluded (found in 1 of the 12 counties) but public hospitals providing first referral level care (also called secondary level) within these counties and estimated to have at least 1000 paediatric admissions per year were considered eligible. One hospital was then purposefully selected from each county except in the largest urban county serving a population of over 3 million from which two hospitals were included. This resulted in two geographic clusters of hospitals (Fig. 1).

These hospitals were invited to join the proposed clinical information network (CIN) after its nature and purpose was explained to each hospitals’ management team and paediatric departments and their agreement sought. Characteristics of hospitals in the CIN are presented in Table 1.

In brief, the CIN collects core data that should be part of the routine health information system (RHIS) (the RHIS dataset) on all children admitted to hospital after their discharge (see below). In 10 of the 12 hospitals a comprehensive set of additional data were also collected on all admissions but due to high workloads in two hospitals (A and C) such comprehensive data were collected only on a random sample of 35% and 65% of the admissions respectively.

### Study population

The population of interest for this report is restricted to children aged 2–59 months, the subjects of available, evidence-based clinical guidelines [9]. Hospitals joined the network in a staggered fashion from September 2013 over a period of 6 months, the period to March 2015 therefore provides at least one full calendar year for analysis from each hospital. In these hospitals, diagnoses at the time of discharge or death are clinician defined and informed by access to only very basic diagnostics (for example malaria blood slide but not blood culture) and post-mortems are done extremely rarely.

### Data collection

Hospitals were encouraged to fully implement a structured pediatric admission record (PAR) that has been shown to improve documentation of core clinical characteristics at admission [10] and which was adopted by the Ministry of Health in 2010 as the admission encounter form for district hospitals [11]. Data were collected from the routine case record in an approach described in full elsewhere [12]. In brief, data were abstracted on the day following discharge from each child’s case record and entered directly onto a computer in a REDCap [13] database with in-built range and validity checks. Data clerks were trained centrally as a team prior to data collection in hospitals according to detailed, written standard operating procedures provided as a manual. At the end of every day before data were synchronized to a central database, the clerk checked on-site for errors, completeness and consistency with locally executed ‘cleaning’ programmes. Any inconsistencies or errors identified were corrected after verification from the case record. Throughout the study period clerks were coordinated and supervised by a research team member who telephoned approximately weekly and made visits approximately two-monthly when ongoing training to improve data collection was conducted.

### Analysis

The Routine Health Information System (RHIS) dataset includes patient age, sex, weight, diagnoses and outcome. Secondary variables described below were generated from these. Age was categorised into 2–6, 7–11, and 12–59 months groups based on differences in observed risks of death in the dataset. The number of diagnoses made at admission (comorbidities) was categorised into no comorbidity, one comorbidity, two comorbidities and three or more comorbidities. Weight-for-age z-score (WAZ) measurement was classified according to standard WHO reference tables for age, weight and gender as follows: children with a Z score, > − 1 were classified as normal, children with a Z score < = − 1 and > −2 were classified as mildly malnourished, children with a Z score, <= −2 and > −3 as moderately malnourished, and children with a Z score < = − 3 as severely malnourished. The RHIS dataset was used to explore those associations between patient characteristics and mortality made possible if the routine health information system were functioning well. A secondary variable was generated to categorise hospital groups by malaria prevalence as an admission diagnosis (high >50%; low <20%). This grouping has some association with HIV prevalence and poverty levels (Table 1).

The comprehensive dataset included an additional checklist of clinical symptoms and signs recorded by the duty clinician on the paediatric admission record. These include danger signs and other signs promoted by Integrated Management of Childhood Illness guidelines [14] and those previously associated with all-cause or disease-specific mortality [15–19] that are used in national [20] and World Health Organisation [21] evidence-based guidelines to guide diagnosis, severity classification and treatment for the commonest causes of paediatric hospital admission (malaria, pneumonia, diarrhoea/dehydration, malnutrition, meningitis, anaemia and asthma). They are listed in Table 2. As there were few observations in each of the V (2%), P (4%) and U (<1%) categories of the AVPU scale these observations were re-categorised into Alert and Not alert.

**Table 2:** Available data by risk factor and hospital

		A	B	C	D	E	F	G	H	I	J	K	L	All hospitals
Observations available		4757	1853	3517	2445	1982	2440	1881	2146	4175	2209	3066	3270	33,741
Routine health information system variables														
Age group														
	2 _6 months	964 (20·3)	386 (20·8)	771 (21·9)	583 (23·8)	247 (12·5)	427 (17·5)	253 (13·5)	243 (11·3)	478 (11·4)	195 (8·8)	343 (11·2)	457 (14·0)	5347 (15·8)
	7 _11 months	1279 (26·9)	488 (26·3)	859 (24·4)	645 (26·4)	474 (23·9)	634 (26·0)	386 (20·5)	340 (15·8)	575 (13·8)	286 (12·9)	472 (15·4)	679 (20·8)	7117 (21·1)
	12_59 months	2514 (52·8)	979 (52·8)	1887 (53·7)	1217 (49·8)	1261 (63·6)	1379 (56·5)	1242 (66·0)	1563 (72·8)	3122 (74·8)	1728 (78·2)	2251 (73·4)	2134 (65·3)	21,277 (63·1)
Child sex														
	Female	2103 (44·2)	763 (41·2)	1563 (44·4)	1083 (44·3)	849 (42·8)	1001 (41·0)	839 (44·6)	939 (43·8)	1912 (45·8)	932 (42·2)	1355 (44·2)	1501 (45·9)	14,840 (44·0)
	Male	2650 (55·7)	1060 (57·2)	1926 (54·8)	1344 (55·0)	1124 (56·7)	1437 (58·9)	981 (52·2)	1186 (55·3)	2194 (52·6)	1204 (54·5)	1701 (55·5)	1766 (54·0)	18,573 (55·0)
	Missing	4 (0·1)	30 (1·6)	28 (0·8)	18 (0·7)	9 (0·5)	2 (0·1)	61 (3·2)	21 (1·0)	69 (1·7)	73 (3·3)	10 (0·3)	3 (0·1)	328 (1·0)
WAZ score														
	0 or > = 1 SD	1852 (38·9)	702 (37·9)	1262 (35·9)	827 (33·8)	1014 (51·2)	1052 (43·1)	1061 (56·4)	1100 (51·3)	2281 (54·6)	1217 (55·1)	1517 (49·5)	1916 (58·6)	15,801 (46·8)
	minus 1 SD	1120 (23·5)	415 (22·4)	630 (17·9)	483 (19·8)	430 (21·7)	534 (21·9)	406 (21·6)	332 (15·5)	697 (16·7)	334 (15·1)	482 (15·7)	619 (18·9)	6482 (19·2)
	minus 2 SD	759 (16·0)	255 (13·8)	488 (13·9)	388 (15·9)	274 (13·8)	331 (13·6)	196 (10·4)	176 (8·2)	384 (9·2)	171 (7·7)	272 (8·9)	304 (9·3)	3998 (11·8)
	minus 3 SD	859 (18·1)	264 (14·2)	611 (17·4)	625 (25·6)	231 (11·7)	414 (17·0)	207 (11·0)	139 (6·5)	326 (7·8)	146 (6·6)	272 (8·9)	254 (7·8)	4348 (12·9)
	Missing	167 (3·5)	217 (11·7)	526 (15·0)	122 (5·0)	33 (1·7)	109 (4·5)	11 (0·6)	399 (18·6)	487 (11·7)	341 (15·4)	523 (17·1)	177 (5·4)	3112 (9·2)
Number of comorbidities														
	0	1800 (37·8)	910 (49·1)	1695 (48·2)	997 (40·8)	1035 (52·2)	824 (33·8)	1042 (55·4)	819 (38·2)	657 (15·7)	968 (43·8)	1131 (36·9)	868 (26·5)	12,746 (37·8)
	1	1776 (37·3)	657 (35·5)	1187 (33·8)	816 (33·4)	640 (32·3)	858 (35·2)	582 (30·9)	782 (36·4)	1665 (39·9)	849 (38·4)	1013 (33·0)	1286 (39·3)	12,111 (35·9)
	2	876 (18·4)	232 (12·5)	471 (13·4)	433 (17·7)	228 (11·5)	497 (20·4)	201 (10·7)	396 (18·5)	1252 (30·0)	298 (13·5)	610 (19·9)	693 (21·2)	6187 (18·3)
	> = 3	305 (6·4)	54 (2·9)	164 (4·7)	199 (8·1)	79 (4·0)	261 (10·7)	56 (3·0)	149 (6·9)	601 (14·4)	94 (4·3)	312 (10·2)	423 (12·9)	2697 (8·0)
Mortality														
	Alive	4535 (95·3)	1744 (94·1)	3227 (91·8)	2182 (89·2)	1932 (97·5)	2326 (95·3)	1838 (97·7)	2007 (93·5)	3901 (93·4)	2098 (95·0)	2854 (93·1)	3060 (93·6)	31,704 (94·0)
	Dead	222 (4·7)	109 (5·9)	290 (8·2)	263 (10·8)	50 (2·5)	114 (4·7)	43 (2·3)	139 (6·5)	274 (6·6)	111 (5·0)	212 (6·9)	210 (6·4)	2037 (6·0)
Comprehensive dataset - Signs and symptoms														
Observations available		2081	1685	1989	2217	1774	2215	1726	1767	3812	1729	2454	2875	26,324
History of fever														
	Missing	25 (1·2)	71 (4·2)	78 (3·9)	62 (2·8)	45 (2·5)	57 (2·6)	38 (2·2)	220 (12·5)	269 (7·1)	99 (5·7)	84 (3·4)	25 (0·9)	1073(4·1)
	No	508 (24·4)	440 (26·1)	405 (20·4)	474 (21·4)	533 (30·0)	567 (25·6)	552 (32·0)	148 (8·4)	374 (9·8)	173 (10·0)	267 (10·9)	597 (20·8)	5038(19·1)
	Yes	1548 (74·4)	1174 (69·7)	1506 (75·7)	1681 (75·8)	1196 (67·4)	1591 (71·8)	1136 (65·8)	1399 (79·2)	3169 (83·1)	1457 (84·3)	2103 (85·7)	2253 (78·4)	20,213(76·8)
History of diarrhoea														
	Missing	40 (1·9)	100 (5·9)	93 (4·7)	87 (3·9)	138 (7·8)	95 (4·3)	47 (2·7)	401 (22·7)	429 (11·3)	212 (12·3)	121 (4·9)	19 (0·7)	1782(6·8)
	No	1083 (52·0)	1021 (60·6)	1051 (52·8)	1177 (53·1)	1174 (66·2)	1374 (62·0)	1243 (72·0)	718 (40·6)	2051 (53·8)	997 (57·7)	1430 (58·3)	1530 (53·2)	14,849(56·4)
	Yes	958 (46·0)	564 (33·5)	845 (42·5)	953 (43·0)	462 (26·0)	746 (33·7)	436 (25·3)	648 (36·7)	1332 (34·9)	520 (30·1)	903 (36·8)	1326 (46·1)	9693(36·8)
History of convulsions														
	Missing	60 (2·9)	118 (7·0)	104 (5·2)	120 (5·4)	133 (7·5)	156 (7·0)	52 (3·0)	429 (24·3)	452 (11·9)	215 (12·4)	170 (6·9)	49 (1·7)	2058(7·8)
	No	1728 (83·0)	1199 (71·2)	1569 (78·9)	1770 (79·8)	1268 (71·5)	1637 (73·9)	1317 (76·3)	955 (54·0)	2332 (61·2)	841 (48·6)	1653 (67·4)	2332 (81·1)	18,601(70·7)
	Yes	293 (14·1)	368 (21·8)	316 (15·9)	327 (14·7)	373 (21·0)	422 (19·1)	357 (20·7)	383 (21·7)	1028 (27·0)	673 (38·9)	631 (25·7)	494 (17·2)	5665(21·5)
History of vomiting every thing														
	Missing	47 (2·3)	53 (3·4)	17 (0·9)	45 (2·1)	15 (0·9)	77 (3·6)	23 (1·4)	354 (24·9)	335 (9·6)	244 (15·6)	69 (3·0)	34 (1·2)	1313(5·3)
	No	1602 (78·4)	1165 (73·8)	1426 (75·3)	1513 (71·6)	1266 (77·0)	1691 (79·7)	1389 (82·3)	765 (53·7)	2074 (59·4)	904 (57·9)	1538 (65·9)	2221 (77·7)	17,554(70·9)
	Yes	395 (19·3)	361 (22·9)	452 (23·9)	555 (26·3)	363 (22·1)	353 (16·6)	275 (16·3)	305 (21·4)	1083 (31·0)	414 (26·5)	727 (31·1)	602 (21·1)	5885(23·8)
Indrawing														
	Missing	46 (2·2)	64 (3·8)	80 (4·0)	107 (4·8)	62 (3·5)	89 (4·0)	25 (1·4)	724 (41·0)	885 (23·2)	541 (31·3)	149 (6·1)	115 (4·0)	2887(11·0)
	No	1306 (62·8)	993 (58·9)	1124 (56·5)	1129 (50·9)	937 (52·8)	1211 (54·7)	1334 (77·3)	873 (49·4)	2527 (66·3)	936 (54·1)	1904 (77·6)	2065 (71·8)	16,339(62·1)
	Yes	729 (35·0)	628 (37·3)	784 (39·4)	981 (44·2)	775 (43·7)	915 (41·3)	367 (21·3)	170 (9·6)	400 (10·5)	252 (14·6)	401 (16·3)	695 (24·2)	7097(27·0)
Pallor														
	Missing	50 (2·4)	127 (7·5)	113 (5·7)	117 (5·3)	152 (8·6)	91 (4·1)	67 (3·9)	211 (11·9)	207 (5·4)	110 (6·4)	131 (5·3)	73 (2·5)	1449(5·5)
	None	1834 (88·1)	1436 (85·2)	1716 (86·3)	1617 (72·9)	1572 (88·6)	1929 (87·1)	1615 (93·6)	1030 (58·3)	2523 (66·2)	1263 (73·0)	1561 (63·6)	2510 (87·3)	20,606(78·3)
	Some/severe	197 (9·5)	122 (7·2)	160 (8·0)	483 (21·8)	50 (2·8)	195 (8·8)	44 (2·5)	526 (29·8)	1082 (28·4)	356 (20·6)	762 (31·1)	292 (10·2)	4269(16·2)
Central cyanosis														
	Missing	62 (3·0)	71 (4·2)	82 (4·1)	74 (3·3)	66 (3·7)	90 (4·1)	24 (1·4)	357 (20·2)	255 (6·7)	199 (11·5)	117 (4·8)	38 (1·3)	1435(5·5)
	No	2015 (96·8)	1606 (95·3)	1890 (95·1)	2118 (95·5)	1684 (94·9)	2111 (95·3)	1695 (98·2)	1406 (79·6)	3537 (92·8)	1523 (88·1)	2324 (94·7)	2803 (97·5)	24,712(93·9)
	Yes	4 (0·2)	8 (0·5)	16 (0·8)	25 (1·1)	24 (1·4)	14 (0·6)	7 (0·4)	4 (0·2)	20 (0·5)	7 (0·4)	13 (0·5)	34 (1·2)	176(0·7)
AVPU														
	Missing	82 (3·9)	128 (7·6)	97 (4·9)	135 (6·1)	76 (4·3)	96 (4·3)	41 (2·4)	317 (18·0)	241 (6·3)	393 (22·8)	160 (6·5)	67 (2·3)	1833(7·0)
	Alert	1901 (91·4)	1449 (86·1)	1693 (85·2)	1932 (87·1)	1643 (92·8)	2021 (91·6)	1640 (95·0)	1359 (77·0)	3358 (88·1)	1171 (67·9)	2074 (84·5)	2640 (91·8)	22,881(87·0)
	Not alert	96 (4·6)	106 (6·3)	196 (9·9)	150 (6·8)	51 (2·9)	90 (4·1)	45 (2·6)	90 (5·1)	212 (5·6)	161 (9·3)	219 (8·9)	168 (5·8)	1584(6·0)
Ability to drink														
	Missing	162 (7·8)	175 (10·4)	102 (5·1)	190 (8·6)	112 (6·3)	219 (9·9)	92 (5·3)	867 (49·1)	1043 (27·4)	639 (37·0)	258 (10·5)	241 (8·4)	4100(15·6)
	No	241 (11·6)	225 (13·4)	468 (23·5)	542 (24·4)	405 (22·8)	217 (9·8)	160 (9·3)	171 (9·7)	206 (5·4)	260 (15·0)	422 (17·2)	307 (10·7)	3624(13·8)
	Yes	1678 (80·6)	1285 (76·3)	1419 (71·3)	1485 (67·0)	1257 (70·9)	1779 (80·3)	1474 (85·4)	729 (41·3)	2563 (67·2)	830 (48·0)	1774 (72·3)	2327 (80·9)	18,600(70·7)
Stiff neck														
	Missing	134 (6·4)	157 (9·3)	114 (5·7)	96 (4·3)	105 (5·9)	109 (4·9)	62 (3·6)	317 (17·9)	367 (9·6)	464 (26·8)	214 (8·7)	47 (1·6)	2186(8·3)
	No/soft	1911 (91·8)	1471 (87·3)	1811 (91·1)	1992 (89·9)	1640 (92·4)	2050 (92·6)	1636 (94·8)	1431 (81·0)	3399 (89·2)	1236 (71·5)	2203 (89·8)	2770 (96·3)	23,550(89·5)
	Yes	36 (1·7)	57 (3·4)	64 (3·2)	129 (5·8)	29 (1·6)	56 (2·5)	28 (1·6)	19 (1·1)	46 (1·2)	29 (1·7)	37 (1·5)	58 (2·0)	588(2·2)
Skin pinch														
	Missing	165 (7·9)	263 (15·6)	110 (5·5)	250 (11·3)	191 (10·8)	232 (10·5)	94 (5·4)	867 (49·1)	1125 (29·5)	654 (37·8)	345 (14·1)	233 (8·1)	4529(17·2)
	Immediate	1568 (75·3)	1087 (64·5)	1657 (83·3)	1281 (57·8)	1260 (71·0)	1620 (73·1)	1461 (84·6)	609 (34·5)	1953 (51·2)	777 (44·9)	1566 (63·8)	2080 (72·3)	16,919(64·3)
	1–2 s	232 (11·1)	237 (14·1)	186 (9·4)	442 (19·9)	279 (15·7)	286 (12·9)	132 (7·6)	203 (11·5)	595 (15·6)	229 (13·2)	445 (18·1)	398 (13·8)	3664(13·9)
	more than or equal to 2 s	116 (5·6)	98 (5·8)	36 (1·8)	244 (11·0)	44 (2·5)	77 (3·5)	39 (2·3)	88 (5·0)	139 (3·6)	69 (4·0)	98 (4·0)	164 (5·7)	1212(4·6)
Capillary refill time														
	Missing	271 (13·0)	268 (15·9)	140 (7·0)	377 (17·0)	496 (28·0)	234 (10·6)	517 (30·0)	1149 (65·0)	1738 (45·6)	710 (41·1)	601 (24·5)	283 (9·9)	6784(25·8)
	<=2 scs	1697 (81·6)	1196 (71·0)	1765 (88·7)	1648 (74·4)	567 (32·0)	1670 (75·4)	1196 (69·3)	494 (28·0)	1917 (50·3)	896 (51·9)	1684 (68·6)	2276 (80·0)	17,006(64·7)
	> = 2 s	108 (5·2)	128 (7·6)	83 (4·2)	185 (8·3)	19 (1·1)	150 (6·8)	11 (0·6)	112 (6·3)	111 (2·9)	96 (5·6)	161 (6·6)	279 (9·8)	1443(5·5)
	Indeterminate	4 (0·2)	93 (5·5)	1 (0·1)	6 (0·3)	692 (39·0)	161 (7·3)	2 (0·1)	12 (0·7)	46 (1·2)	26 (1·5)	8 (0·3)	7 (0·2)	1058(4·0)
Sunken eyes														
	Missing	474 (22·8)	490 (29·1)	148 (7·4)	329 (14·8)	126 (7·1)	236 (10·7)	49 (2·8)	771 (43·6)	1012 (26·5)	615 (35·6)	221 (9·0)	159 (5·5)	4630(17·6)
	No	1303 (62·6)	947 (56·2)	1662 (83·6)	1408 (63·5)	1374 (77·5)	1687 (76·2)	1493 (86·5)	724 (41·0)	2289 (60·0)	989 (57·2)	1885 (76·8)	2155 (75·0)	17,916(68·1)
	Yes	304 (14·6)	248 (14·7)	179 (9·0)	480 (21·7)	274 (15·4)	292 (13·2)	184 (10·7)	272 (15·4)	511 (13·4)	125 (7·2)	348 (14·2)	561 (19·5)	3778(14·4)
Jaundice														
	Missing	27 (1·3)	72 (4·3)	95 (4·8)	73 (3·3)	122 (6·9)	64 (2·9)	33 (1·9)	215 (12·2)	131 (3·4)	96 (5·6)	100 (4·1)	34 (1·2)	1062(4·0)
	none	2044 (98·2)	1593 (94·5)	1881 (94·6)	2102 (94·8)	1645 (92·7)	2136 (96·4)	1679 (97·3)	1486 (84·1)	3557 (93·3)	1593 (92·1)	2171 (88·5)	2802 (97·5)	24,689(93·8)
	Moderate/severe	10 (0·5)	20 (1·2)	12 (0·6)	42 (1·9)	7 (0·4)	15 (0·7)	14 (0·8)	66 (3·7)	124 (3·3)	40 (2·3)	183 (7·5)	39 (1·4)	572(2·2)
Severe wasting														
	Missing	155 (7·4)	149 (8·8)	106 (5·3)	201 (9·1)	123 (6·9)	271 (12·2)	43 (2·5)	856 (48·4)	1180 (31·0)	639 (37·0)	195 (7·9)	221 (7·7)	4139(15·7)
	No	1804 (86·7)	1455 (86·4)	1782 (89·6)	1686 (76·0)	1609 (90·7)	1781 (80·4)	1622 (94·0)	856 (48·4)	2542 (66·7)	1042 (60·3)	2141 (87·2)	2555 (88·9)	20,875(79·3)
	Yes	122 (5·9)	81 (4·8)	100 (5·0)	330 (14·9)	42 (2·4)	163 (7·4)	61 (3·5)	55 (3·1)	90 (2·4)	48 (2·8)	118 (4·8)	99 (3·4)	1309(5·0)
Oedema of malnutrition														
	Missing	128 (6·2)	108 (6·4)	98 (4·9)	178 (8·0)	139 (7·8)	201 (9·1)	44 (2·5)	533 (30·2)	408 (10·7)	296 (17·1)	207 (8·4)	45 (1·6)	2385(9·1)
	none	1924 (92·5)	1538 (91·3)	1863 (93·7)	1895 (85·5)	1624 (91·5)	1980 (89·4)	1672 (96·9)	1207 (68·3)	3350 (87·9)	1416 (81·9)	2097 (85·5)	2765 (96·2)	23,331(88·6)
	Moderate/severe	29 (1·4)	38 (2·3)	27 (1·4)	144 (6·5)	11 (0·6)	34 (1·5)	10 (0·6)	27 (1·5)	54 (1·4)	17 (1·0)	150 (6·1)	65 (2·3)	606(2·3)
Auxilliary variables for multiple imputation														
PAR														
	PAR used	815 (60·4)	855 (80·0)	745 (68·0)	939 (68·3)	1088 (98·0)	1419 (98·1)	1130 (99·4)	669 (68·1)	2053 (89·7)	911 (91·5)	1136 (82·3)	1373 (85·7)	13,133(83·0)
	PAR present not used	535 (39·6)	206 (19·3)	351 (32·0)	336 (24·4)	7 (0·6)	5 (0·3)	3 (0·3)	1 (0·1)	151 (6·6)	70 (7·0)	169 (12·2)	162 (10·1)	1996(12·6)
	No PAR	0 (0·0)	8 (0·7)	0 (0·0)	100 (7·3)	15 (1·4)	22 (1·5)	4 (0·4)	312 (31·8)	84 (3·7)	15 (1·5)	75 (5·4)	68 (4·2)	703(4·4)
History of cough														
	Missing	28 (1·3)	73 (4·3)	87 (4·4)	74 (3·3)	52 (2·9)	73 (3·3)	42 (2·4)	452 (25·6)	553 (14·5)	217 (12·6)	121 (4·9)	23 (0·8)	1795(6·8)
	No	736 (35·4)	630 (37·4)	737 (37·1)	617 (27·8)	583 (32·9)	717 (32·4)	681 (39·5)	516 (29·2)	1651 (43·3)	648 (37·5)	1045 (42·6)	1344 (46·7)	9905(37·6)
	Yes	1317 (63·3)	982 (58·3)	1165 (58·6)	1526 (68·8)	1139 (64·2)	1425 (64·3)	1003 (58·1)	799 (45·2)	1608 (42·2)	864 (50·0)	1288 (52·5)	1508 (52·5)	14,624(55·6)
History of difficulty breathing														
	Missing	39 (1·9)	102 (6·1)	105 (5·3)	95 (4·3)	80 (4·5)	137 (6·2)	60 (3·5)	637 (36·0)	810 (21·2)	341 (19·7)	152 (6·2)	36 (1·3)	2594(9·9)
	No	1191 (57·2)	867 (51·5)	1199 (60·3)	918 (41·4)	1065 (60·0)	1301 (58·7)	1164 (67·4)	855 (48·4)	2272 (59·6)	962 (55·6)	1737 (70·8)	2147 (74·7)	15,678(59·6)
	Yes	851 (40·9)	716 (42·5)	685 (34·4)	1204 (54·3)	629 (35·5)	777 (35·1)	502 (29·1)	275 (15·6)	730 (19·2)	426 (24·6)	565 (23·0)	692 (24·1)	8052(30·6)

Variables included in the study stratified by routine health information system (RHIS) variables, comprehensive dataset (includes clinical risk factors) and auxiliary variables (included only in the imputation model). Data available and missing at hospital and overall are presented as numbers and proportions

### Statistical analysis

**Fig. 2:**
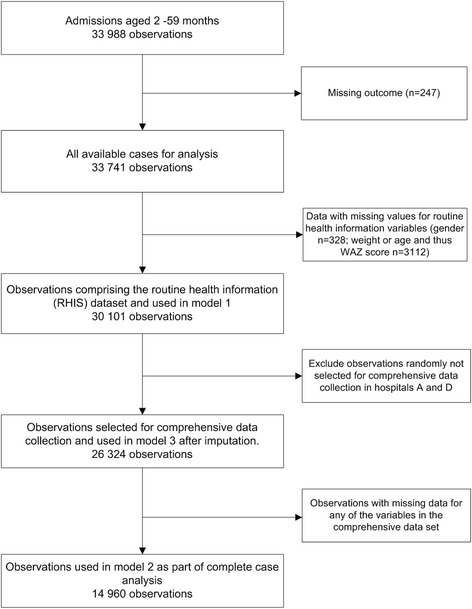
Availability of data across the different models. Illustrates the number of observations used in each of the models listed

All patients without outcome data or with an implausible or missing date of admission, discharge or death were dropped from the analysis (see Fig. 2). Using the RHIS dataset we present hospital specific crude mortality rates and accompanying confidence intervals. To explore whether hospital mortality was associated with hospital identity, we used the RHIS dataset and fitted a fixed effects model with hospital but no other covariates and compared this to a null model using a likelihood ratio test (LRT). Hospitals were retained as fixed effects in all multivariable models because of significant associations with mortality and because we had only a small, non-random sample of hospitals (considering hospitals identities as random effects made no appreciable difference to results, data not shown).

The data available on clinical signs, symptoms and diagnoses are presented as hospital specific proportions in Table 2 and illustrate the variability across sites. We used logistic regression without adjustment to explore associations with mortality for each variable but without hospitals as fixed effects (Table *3*). We then built multivariable models to explore associations of key clinical factors with mortality. We included malaria prevalence as a fixed effect in the multivariable models and explored for interaction between the risk factors and malaria prevalence. We used a multivariable model (model 1) based on the RHIS dataset to explore the ability of these data to explain mortality (Table 2). Model 2 included all variables in the comprehensive dataset (Table 2) in a complete case analysis including interaction terms for malaria prevalence. This included only 57% of all cases as a result of list-wise deletion of records with any missing data (see Fig. 2). The degree of missingness, represented as a proportion, varied by hospital and variable (Table 2). To address the missing data problem multiple imputation was used (panel 2) with the validity of results explored in sensitivity analyses (panel 3).

**Table 3:** Univariable and Multivariable analysis for associations with mortality

		Univariable analysis - pooled data		Model 2 - Mulitvariable model for associations with mortality for complete case analysis including interaction terms for malaria endemicity	Model 3 - Mulitvariable model for associations with mortality on imputed data including interaction terms for malaria endemicity
		OR (95% CI)	P value	OR (95% CI)	OR (95% CI)
AUC				0·86	0·85
Pseudo R-squared				0·25	0·24
Age group^a^					
	2 _6 months	ref			
	7 _11 months	0·72(0·64–0·82)	<0·001		
	12_59 months	0·40(0·36–0·45)			
	2_6 months in high malaria prevalence			ref	ref
	7 _11 months in high malaria prevalence			0·46(0·36–0·60)	0·43(0·29–0·63)
	12_59 months in high malaria prevalence			0·41(0·33–0·50)	0·37(0·27–0·50)
	7 _11 months in Low malaria prevalence			0·34(0·15–0·75)	0·33(0·19–0·57)
	12_59 months in Low malaria prevalence			0·29(0·14–0·62)	0·29(0·17–0·49)
Child sex					
	Female	ref		ref	ref
	Male	0·79(0·72–0·86)	<0·001	0·71(0·64–0·80)	0·67(0·57–0·79)
WAZ score^a^					
	0 or > = 1 SD	ref			
	Minus 1 SD	1·35(1·17–1·56)	<0·001		
	Minus 2 SD	2·26(1·95–2·61)			
	Minus 3 SD	4·35(3·86–4·92)			
	0 or > = 1 SD in high malaria prevalence			ref	ref
	Minus 1 SD in high malaria prevalence			1·50(1·15–1·95)	1·19(0·85–1·67)
	Minus 2 SD in high malaria prevalence			1·25(0·95–1·65)	1·50(1·03–2·17)
	Minus 3 SD in high malaria prevalence			2·96(2·27–3·86)	3·01(2·09–4·35)
	Minus 1 SD in Low malaria prevalence			0·93(0·45–1·93)	1·06(0·61–1·82)
	Minus 2 SD in Low malaria prevalence			1·17 (0·56–2·44)	0·88(0·52–1·51)
	Minus 3 SD in Low malaria prevalence			2·36(1·13–4·94)	2·09(1·22–3·56)
Number of comorbidities					
	0	ref		ref	ref
	1	1·44(1·28–1·62)	<0·001	0·99(0·85–1·16)	1·08(0·86–1·37)
	2	2·21(1·94–2·50)		1·03(0·86–1·23)	1·27(0·98–1·64)
	> = 3	3·67(3·18–4·23)		1·25(1·02–1·55)	1·68(1·25–2·25)
History of fever					
	No	ref		ref	ref
	Yes	0·96(0·84–1·10)	0·555	0·77(0·66–0·90)	0·76(0·62–0·92)
History of diarrhoea					
	No	ref		ref	ref
	Yes	2·02(1·82–2·25)	<0·001	1·34(1·16–1·56)	1·45(1·20–1·76)
Convulsions					
	No	ref		ref	ref
	Yes	1·13(1·00–1·28)	0·045	1·54(1·32–1·81)	1·35(1·09–1·67)
Vomitting everything					
	No	ref		ref	ref
	Yes	1·41(1·25–1·58)	<0·001	1·04(0·91–1·20)	1·03(0·86–1·24)
Indrawing					
	No	ref		ref	ref
	Yes	2·84(2·55–3·18)	<0·001	2·61(2·28–2·98)	2·48(2·08–2·96)
Pallor					
	None	ref		ref	ref
	Some/severe	3·39(3·03–3·79)	<0·001	2·21(1·93–2·53)	2·32(1·92–2·81)
Central cyanosis					
	No	ref		ref	ref
	Yes	6·36(4·54–8·90)	<0·001	2·64(1·70–4·12)	3·25(1·91–5·53)
AVPU					
	Alert	ref		ref	ref
	Not alert (VPU)	8·95(7·88–10·17)	<0·001	3·98(3·31–4·77)	3·95(3·16–4·95)
Ability to drink^a^					
	No	ref			
	Yes	0·23(0·20–0·26)	<0·001		
	Ability to drink no in high malaria prevalence			ref	ref
	Ability to drink yes in high malaria prevalence			0·63(0·49–0·80)	0·65(0·48–0·87)
	Ability to drink yes in Low malaria prevalence			0·51(0·24–1·08)	0·44(0·25–0·78)
Stiff neck^a^					
	No/soft	ref			
	Yes	2·71(2·13–3·44)	<0·001		
	Stiff neck no in high malaria prevalence			ref	ref
	Stiff neck yes in high malaria prevalence			2·17(1·38–3·42)	3·92(2·15–7·16)
	Stiff neck yes in low malaria prevalence			3·07(1·28–7·36)	1·53(0·81–2·90)
Skin pinch					
	Immediate	ref		ref	ref
	1–2 s	2·25(1·96–2·59)	<0·001	1·29(1·09–1·52)	1·17(0·94–1·44)
	> 2 s	6·18(5·28–7·24)		1·80(1·43–2·26)	1·47(1·09–1·98)
Capillary refill time					
	<=2 scs	ref		ref	ref
	>3 s	3·28(2·80–3·84)	<0·001	1·46(1·15–1·86)	1·68(1·32–2·13)
	Indeterminate	0·70(0·51–0·97)		1·17(0·79–1·73)	1·83(1·12–2·99)
Sunken eyes					
	No	ref		ref	ref
	Yes	2·76(2·44–3·12)	<0·001	1·12(0·96–1·31)	1·24(1·00–1·55)
Jaundice					
	None	ref		ref	ref
	Moderate/severe	2·03(1·55–2·66)	<0·001	1·78(1·30–2·42)	1·64(1·01–2·65)
Severe wasting^a^					
	No	ref			
	Yes	5·01(4·32–5·81)	<0·001		
	Severe wasting no in high malaria prevalence			ref	ref
	Severe wasting yes in high malaria prevalence			2·35(1·68–3·27)	2·54(1·70–3·82)
	Severe wasting yes in low malaria prevalence			1·99(0·97–4·09)	1·66(0·95–2·87)
Oedema of malnutrition^a^					
	None	ref			
	Moderate/severe	3·02(2·39–3·80)	<0·001		
	Oedema none in high malaria prevalence			ref	ref
	Oedema mild/moderate in high malaria prevalence			2·66(1·82–3·89)	3·13(1·95–5·02)
	Oedema mild/moderate in Low malaria prevalence			2·45(1·17–5·13)	1·88(1·08–3·25)
Malaria endemicity					
	High	ref	<0·001	ref	ref
	Low	0·90(0·83–0·99)		0·71(0·46–1·08)	0·78(0·43–1·42)
Hospital					
	A	ref		ref	ref
	B	1·23(1·01–1·61)	<0·001	1·26(0·91–1·73)	1·69(1·04–2·74)
	C	1·83(1·53–2·20)		1·74(1·30–2·32)	1·90(1·26–2·85)
	D	2·46(2·04–2·96)		1·53(1·16–2·01)	1·99(1·33–2·98)
	E	0·53(0·39–0·72)		0·60(0·40–0·90)	0·53(0·28–1·00)
	F	1·00(0·79–1·26)		0·91(0·66–1·26)	0·88(0·56–1·39)
	G	0·48(0·34–0·67)		0·75(0·49–1·15)	0·81(0·43–1·50)
	H	1·41(1·14–1·76)		0·83(0·62–1·11)	0·90(0·52–1·57)
	I	1·43(1·20–1·72)		1·23(0·98–1·55)	1·15(0·82–1·60)
	J	1·08(0·86–1·37)		0·68(0·50–0·92)	1·05(0·68–1·63)
	K	1·52(1·25–1·84)		0·82(0·64–1·07)	0·95(0·68–1·32)
	L	1·40(1·15–1·70)		na	na

Model 2 results are based on complete case analysis while model 3 results are based on the imputed dataset; both models include interaction terms for malaria prevalence.

^a^Variables with significant interactions with malaria endemicity

***Panel 2: Handling missing data***

We explored and subsequently assumed a missing at random (MAR) mechanism as a basis for multiple imputation using the chained equation methods proposed by van Buuren [22] and Raghunathan [23]. Imputation was based on 100 iterations and 10 datasets as has been recommended for missing data rates of 10% -30% per variable[24, 25]. All variables in the RHIS and comprehensive datasets and identified interaction terms were included in the imputation procedures. To improve the power of the imputation model, we included auxiliary variables (history of cough and difficulty breathing) which may be clinically useful in diagnosis and are relatively well documented, and a variable denoting use of the paediatric admission record as this improves documentation and may therefore influence missingness [10]. We replicated analyses of associations with mortality including interaction terms after imputation in Model 3 (see Table 3).

In order to assess the plausibility of a MAR mechanism, we performed analysis under a Missing Not At Random (MNAR) assumption using pattern mixture models that included interaction terms. This proceeded as follows; first, we derived three missingness patterns amongst cases in the dataset: no missing data (57% cases); minimum 1 to maximum 3 variables per case with missing data (26% cases); >3 variables per case with missing data (17% cases). We performed multiple imputations and fitted the same multivariable models for each pattern independently. Thereafter, we pooled the estimates across the three patterns weighting by the proportions of individuals in each pattern per variable and compared these results with model 3 estimates estimated under a MAR assumption. We present the results of the MNAR analyses in Appendix.

***Panel 3: Sensitivity analyses***

We conducted various sensitivity analyses to explore the consistency of our results under different scenarios. First, we explored associations with mortality using the comprehensive imputed dataset but restricted the analysis to cases with only common childhood illnesses (malaria, pneumonia, diarrhoea/dehydration, malnutrition, meningitis, anaemia and asthma) to exclude possible effects of uncommon, high mortality conditions that might vary across place. Second, we conducted analyses restricted to the ‘best months’ of data collection by excluding the first 9 months (November 2013 to July 2014) for hospitals I and J and 7 months (February to August 2014) for hospital H to limit the scale of imputation. Third, we undertook analyses for data stratified according to whether cases were in high or low admission seasons. The estimates for associations between risk factors and mortality from these sensitivity analyses were clinically not appreciably different from those reported for Model 3 (**data available on request**).

Calibration and discrimination of the models was assessed using pseudo R-squared and area under the receiver operating curve (AUC) measures. Results from the univariable and multivariable analyses are reported as crude and adjusted odds ratios respectively with corresponding 95% confidence intervals (CIs) adjusted for clustering within hospitals. All analyses were undertaken using Stata v13 (StataCorp, Texas, USA).

## Results

**Fig. 3:**
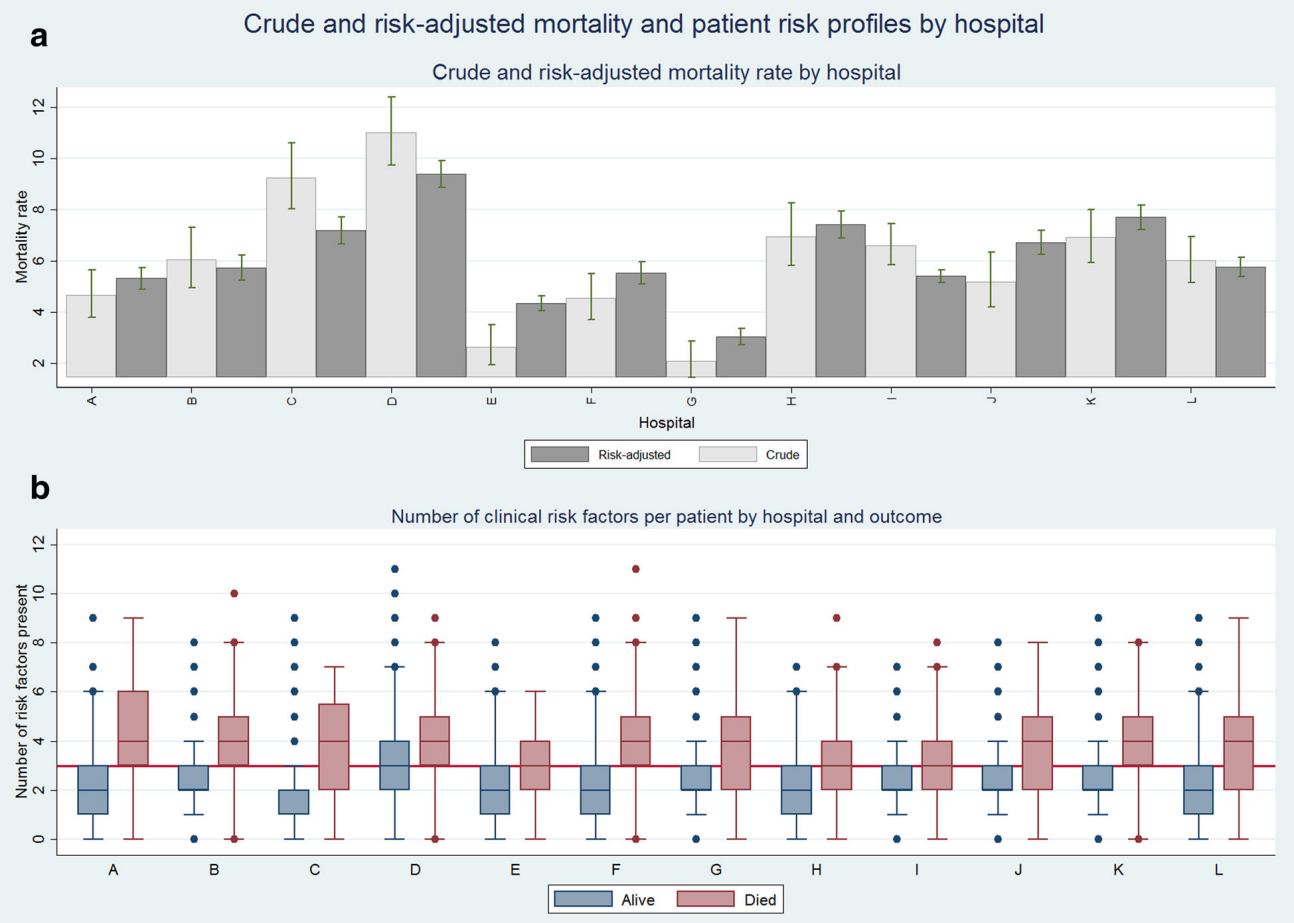
Crude and risk-adjusted mortality and patient risk profiles by hospital. The top panel Fig. 3a represent crude (light grey) and risk-adjusted mortality rate (dark grey) across hospitals with accompanying 95% confidence intervals. The bottom panel Fig. 3b represent the median and the 25th and 75th interquartile ranges for number of risk factors per patient stratified by mortality. Hospitals are ordered by malaria endemicity (low A, B, C, D, E, F, G; high H, I, J, K, L)

A total of 44,314 children were admitted into the CIN hospitals from September 2013 to March 2015, of these 33,741 (76%) were aged 2–59 months, had outcome data and a plausible date of admission or death. Characteristics of children by hospital and overall are presented in Table *2*. The overall cluster adjusted crude mortality across hospitals was 6.2% (95% CI 4.9 to 7.8; range 2.1% - 11.0%) with five-fold variation across hospitals while the risk-adjusted mortality rate derived from Model 3 was 6.2% (95% CI 4.7 to 7.6; range 3.0% - 9.4%) (Fig. 3a). Hospital identity was significantly associated with mortality (likelihood ration test, LRT <0.001 when compared to the null model). The distribution of risk factors per admission varied by hospital and outcome with 85% of children who survived having 3 or fewer risk factors while of those who died 53% had more than 3 risk factors (see Fig. 3b).

The amount of missing data varied by variable and hospital (see Table 2 and panel 2). Pooled estimates of association weighted by proportion across missingness patterns were similar to those from model 3 (imputed data assuming MAR) and are presented in Appendix. These findings provide support for assuming that data were missing at random.

### Clinical risk factors for mortality

All characteristics included in the RHIS dataset, derived covariables and all primary symptoms and signs included in the comprehensive data were significantly associated with mortality in univariable analyses except history of fever with anticipated (Table 3) direction of effect. Although we utilised alert vs not alert in risk adjustment exploratory univariable analysis illustrated an increasing risk of mortality with a decreasing conscious level; with alert as the reference category risks were V OR 4.61; 95% CI 3.54–6.00, P OR 9.31; 95% CI 7.84–11.05 and U OR 17.59; 95% 13.42–23.04.

Multivariable model 1, using RHIS data had an AUC of 0.73 and pseudo-R^2^ of 0.09 suggesting poor to modest model fit (assuming a cut off for good model fit for pseudo-R^2^ of 0.20 and above [26]) and that they are not likely to be suitable for understanding hospital populations’ risk of mortality. The complete case analysis model (model 2) and analyses after imputation (model 3), including malaria prevalence interactions, had better model fit (pseudo-R^2^ of 0.25; AUC of 0.86 and pseudo-R^2^ of 0.24; AUC of 0.85 respectively).

Estimates of association from complete case analysis and data sets using all admissions after imputation were similar in magnitude and direction of effect across all the clinical risk factors and we therefore present results from model 3 using imputed data as this makes maximum use of available data. These analyses (Table 3) show in all cases where interactions were not found that risk factors remained significantly associated with mortality except vomiting everything and number of comorbidities. Male gender (OR 0.67; 95% CI 0.57–0.79) and history of fever (OR 0.76; 95% CI 0.62–0.92) were associated with protection while reduced conscious level was strongly associated with mortality (OR 3.95; 95% CI 3.16–4.95). Tests for interactions between risk factors and malaria prevalence were significant for ability to drink, stiff neck, severe wasting, oedema, age group and weight-for-age z-score.

For covariables with significant interactions, estimates of association for having a stiff neck and severe wasting in low malaria prevalence hospitals had consistent but lower magnitude directions of effect than in high malaria prevalence areas (OR 1.53; 95% CI 0.81 to 2.90 vs 3.92; 95% CI 2.15 to 7.16 and OR 1.66; 95% CI 0.95 to 2.87 vs OR 2.54; 95% CI 1.70–3.82 respectively) and were not significant (Table 3) perhaps reflecting a loss of power (see Table 2). The estimates of association for being able to drink, oedema of malnutrition and age group remained significant in both malaria prevalence settings with consistently lower magnitudes of association in low malaria prevalence settings (OR, 0.65 vs 0.44, 3.13 vs 1.88, and 0.43 vs 0.33 (7–11 months) and 0.37 vs 0.29 (12–59 months) respectively). For weight-for-age z-score the estimate of effect was also attenuated in the low malaria prevalence hospitals (OR 2.09; 95% CI 1.22–3.56 vs OR 3.01; 95% CI 2.09–4.35). Signs associated with dehydration were consistently associated with mortality. For three hospitals (B, C and D) a persistent association with mortality was observed within these multivariable models (ORs 1.69, 1.90 and 1.99 respectively, Table 3).

## Discussion

All-cause mortality is highly variable across only 12 hospitals even within a common age group. Variation in mortality was associated with the proportion of children with multiple risk factors, something that cannot be determined using data from existing routine health information systems. This variation in risk factors at presentation might be linked to the varying number of comorbidities at presentation resulting from overlapping syndromic diagnoses. This finding of major variation in the risk profile of children admitted to hospitals is important but rarely highlighted in existing research literature from low-income settings. The variation of mortality with hospital identity is probably explained by associated variability in underlying risk factors (such as malaria and HIV prevalence, socio-economic status, nutrition and access) that influence case-mix and case-severity. Although, these data exist at a population level, adjusting for these parameters would require a large number of hospitals in more defined, smaller regions. As such we have refrained from adjusting for these parameters in our models due to the risk of ecological fallacy where population estimates do not necessarily apply to the population seeking care. In addition, care seeking patterns in Kenya vary across populations and individuals with some of the patients accessing care directly from hospital while others are through referral from primary health care services but these data are rarely documented and hence difficult to disentangle within this population. Thus, although there is a standard policy guiding the provision of PHC in Kenya and how patients might progress through the system anecdotal evidence suggests procedures are rarely followed in practice and there are limited data from primary care on access and care seeking behaviors.

Mortality may also potentially be influenced by differing availability of resources across hospitals and variation in care practices shown to exist in other studies [27, 28]. More comprehensive datasets from a larger number of hospitals, as are being used in high income settings [29, 30], would however be required to try and determine the degree to which quality of care and local context are associated with inpatient mortality in low-income settings. Even then the use of mortality to infer anything about quality of care specifically is contested [1, 31]. What is more pertinent to low-income contexts is that such variation in mortality should prompt thinking on where system strengthening efforts may be most needed.

In the multivariable analyses, we demonstrate that majority of clinical risk factors included in best-practice guidelines were associated with mortality irrespective of diagnosis. This approach is important due to the inability to confirm diagnoses in these settings. For example, we have previously reported that basic investigations like blood culture, CSF analysis and urine culture are not routinely available [28] and there is no access to tests of inflammatory markers, for biochemical derangements or for detection of other pathogens except for malaria and HIV. Thus, an approach that is agnostic of diagnosis may be the more useful approach at scale. Prior demonstrations of association are typically from single sites or focus on specific diseases [15, 16, 19, 32]. We believe this is the first report of the value of these clinical risk factors from multiple settings, across all cause admissions, with routine observations made by large numbers of clinicians. As malaria prevalence and other attributes of the hospitals’ setting may be correlated, including for example proportion living in poverty and hospital size, we cannot attribute the influence of malaria prevalence on the behaviour of risk factors to malaria as a disease. However, findings suggest that future efforts to explore variability in mortality or develop risk prediction approaches should take the prevalence of malaria into account.

Our data support the practical, day to day use of these clinical factors in identifying children who may be prioritised for attention, specific treatments and review. Integrating such clinical factors into a low-income setting, patient-level prognostic score might be possible [16] replicating approaches in high-income settings [33]. However, the implementation of such scores in routine settings with limited human resources, high staff turnover and without computer assisted decision support would likely be very challenging as even basic job aides are somewhat slowly adopted [28]. These clinical features might however be used to characterise risk profiles of hospital populations aged 2 to 59 months. This might enable improved understanding of changes in mortality over time within sites using methods such as cumulative sum control charts (CUSUM) [34–37] where risk-adjustment may facilitate exploration of variation in a single institution’s performance [1, 3, 31]. More pragmatically such risk profiles may help point to different health system challenges. High prevalence of cases with high risk factor density may suggest problems of access, late detection or delayed referral. They may also point to particularly vulnerable populations within catchment areas, where malnutrition and poverty are prevalent for example, or demonstrate the impact of varying disease ecology that should be taken into account in resource allocation. Our findings suggest that existing routine health information systems data (such as DHIS2) [38] would be inadequate for this purpose, suggesting value in developing and implementing suitable common data frameworks.

The data we report need to be interpreted in the light of their limitations. First, is the missing data problem commonly associated with collecting data in routine settings. The Clinical Information Network worked with hospitals to promote data quality that improved over time but missingness varied across variables and hospitals. Thus, just less than 60% of cases were included in our complete case multivariable models. We used multiple imputation to allow use of all available data and undertook sensitivity analyses that suggest our findings are generally robust. An alternative approach would be using Expectation-Maximization (EM) algorithms to get maximum likelihood estimates [39]. Second, our sample of hospitals is small and non-random, with selection based on feasibility and efforts to represent diverse but not all epidemiological and socio-cultural contexts. One hospital that failed to provide reasonable quality data was excluded from these analyses. As such, due to the limited number of hospitals, our models did not sufficient power to explore potentially important factors at the hospital level. Thirdly, diagnoses are clinical and rarely informed by diagnostics while risk factors such as hypoglycaemia, hypoxemia or an individual’s HIV status could not be examined as these are rarely routinely evaluated. Fourth, we did not include more robust nutritional indicators like mid-upper arm circumference or weight-for-height z scores or account for vaccination status which may influence mortality because such data are largely missing. However, there is evidence that vaccination coverage is high in Kenya and we were able to use weight-for-age z scores for nutritional assessment.

## Conclusion

In summary, all-cause crude and cluster adjusted mortality rate was highly variable across hospitals. Such variation is largely explained by variation in severity of illness at the time of clinical presentation, findings that point to underlying differences in population health and health system performance that will need to be explored. Our data supports the use of clinical risk factors drawn from guidelines in day-to-day use in prioritizing care and identifying children at the highest risk of death but also to develop risk adjusted mortality estimates across hospitals. We also demonstrate how having a large patient level dataset from multiple geographically diverse sites may improve our understanding of health system challenges and performance. Such work provides a learning platform for the design of common data frameworks that are relevant to clinical practice and might be incorporated into future electronic medical records (EMRs) that go beyond a focus on cost-accounting needs [40]. To maximise the future value of EMRs there is a clear imperative for researchers, clinicians, policy makers and health care managers to engage in their design so they enable health system performance monitoring at scale as is occurring in specific fields such as HIV care [41, 42].

## Appendix

**Table 4:** Sensitivity analysis for imputation based on the MAR versus MNAR assumption high malaria prevalence strata

		Variables with no missing data	1–3 variables with missing data	4 or more variables with missing data	Mantel Haenszel estimates weighted across missing patterns (MNAR)	Model 3 - Mulitvariable model for associations with mortality on imputed data including interaction terms for malaria endemicity (MAR)
		OR (95% CI)	OR (95% CI)	OR (95% CI)	OR (95% CI)	OR (95% CI)
Age group^a^						
	2_6 months and high malaria prevalence	ref	ref	ref	ref	ref
	7 _11 months in high malaria prevalence	0·43(0·29–0·63)	0·48(0·27–0·86)	0·47(0·29–0·77)	0·48(0·28–0·81)	0·43(0·29–0·63)
	12_59 months in high malaria prevalence	0·37(0·27–0·5)	0·53(0·34–0·84)	0·35(0·23–0·54)	0·37(0·26–0·51)	0·37(0·27–0·50)
	7 _11 months in Low malaria prevalence	0·34(0·15–0·75)	0·54(0·17–1·68)	0·09(0·02–0·35)	0·41(0·14–1·21)	0·33(0·19–0·57)
	12_59 months in Low malaria prevalence	0·29(·14-·62)	0·59(0·21–1·72)	0·07(0·02–0·25)	0·39(0·15–1·07)	0·29(0·17–0·49)
Child sex						
	Female	ref	ref	ref	ref	ref
	Male	0·67(0·57–0·79)	0·7(0·56–0·88)	0·82(0·63–1·05)	0·71(0·58–0·87)	0·67(0·57–0·79)
WAZ score^a^						
	0 or > = 1 SD in high malaria prevalence	ref	ref	ref	ref	ref
	Minus 1 SD in high malaria prevalence	1·19(0·85–1·67)	1·96(1·25–3·09)	1·54(0·91–2·58)	1·46(0·96–2·23)	1·19(0·85–1·67)
	Minus 2 SD in high malaria prevalence	1·50(1·03–2·17)	1·43(0·81–2·54)	0·89(0·47–1·7)	1·29(0·79–2·12)	1·50(1·03–2·17)
	Minus 3 SD in high malaria prevalence	3·01(2·09–4·35)	5·23(3·21–8·54)	1·76(0·95–3·25)	3·20(2·10–4·91)	3·01(2·09–4·35)
	Minus 1 SD in Low malaria prevalence	0·93(·45–1·94)	2·20(0·8–6·01)	0·29(0·07–1·18)	1·20(0·50–2·95)	1·06(0·61–1·82)
	Minus 2 SD in Low malaria prevalence	1·17(·56–2·44)	1·61(0·55–4·69)	0·17(·04–0·67)	1·18(0·50–2·83)	0·88(0·52–1·51)
	Minus 3 SD in Low malaria prevalence	2·36(1·13–4·94)	5·85(2·07–16·53)	0·33(0·08–1·33)	3·06(1·26–7·65)	2·09(1·22–3·56)
Number of comorbidities						
	0	ref	ref	ref	ref	ref
	1	1·08(0·86–1·37)	1·21(0·89–1·65)	0·78(0·57–1·08)	0·98(0·75–1·28)	1·08(0·86–1·37)
	2	1·27(0·98–1·64)	1·26(0·89–1·79)	0·67(0·45–0·99)	1·19(0·88–1·62)	1·27(0·98–1·64)
	> = 3	1·68(1·25–2·25)	1·22(0·78–1·89)	0·92(0·56–1·53)	1·20(0·80–1·82)	1·68(1·25–2·25)
History of fever						
	No	ref	ref	ref	ref	ref
	Yes	0·76(0·62–0·92)	0·79(0·6–1·05)	0·76(0·5–1·17)	0·77(0·59–1·00)	0·76(0·62–0·92)
History of diarrhoea						
	No	ref	ref	ref	ref	ref
	Yes	1·45(1·2–1·76)	1·05(0·8–1·39)	1·36(0·92–2)	1·32(1·02–1·71)	1·45(1·20–1·76)
convulsions						
	No	ref	ref	ref	ref	ref
	Yes	1·35(1·09–1·67)	1·73(1·29–2·33)	1·67(1·13–2·47)	1·53(1·15–2·06)	1·35(1·09–1·67)
Vomitting everything						
	No	ref	ref	ref	ref	ref
	Yes	1·03(0·86–1·24)	1·07(0·81–1·41)	1·11(0·79–1·57)	1·06(0·83–1·36)	1·03(0·86–1·24)
Indrawing						
	No	ref	ref	ref	ref	ref
	Yes	2·48(2·08–2·96)	2·91(2·27–3·73)	2·58(1·82–3·64)	2·61(2·07–3·31)	2·48(2·08–2·96)
Pallor						
	None	ref	ref	ref	ref	ref
	Some/severe	2·32(1·92–2·81)	2·22(1·71–2·87)	1·98(1·45–2·69)	2·19(1·73–2·79)	2·32(1·92–2·81)
Central cyanosis						
	No	ref	ref	ref	ref	ref
	Yes	3·25(1·91–5·53)	2·37(1·1–5·11)	2·3(0·52–10·21)	2·77(1·36–6·33)	3·25(1·91–5·53)
AVPU						
	Alert	ref	ref	ref	ref	ref
	Not alert (VPU)	3·95(3·16–4·95)	3·94(2·87–5·41)	4·42(2·53–7·73)	4·06(2·93–5·73)	3·95(3·16–4·95)
Ability to drink^a^						
	Ability to drink no in high malaria prevalence	ref	ref	ref	ref	ref
	Ability to drink yes in high malaria prevalence	0·65(0·48–0·87)	0·76(0·5–1·15)	0·58(0·33–1·01)	0·66(0·45–0·98)	0·65(0·48–0·87)
	Ability to drink yes in Low malaria prevalence	0·51(0·24–1·08)	0·85(0·31–2·34)	0·11(0·02–0·48)	0·57(0·23–1·43)	0·44(0·25–0·78)
Stiff neck^a^						
	Stiff neck no in high malaria prevalence	ref	ref	ref	ref	ref
	Stiff neck yes in high malaria prevalence	3·92(2·15–7·16)	2·24(0·94–5·31)	0·81(0·28–2·31)	2·70(1·36–5·49)	3·92(2·15–7·16)
	Stiff neck yes in low malaria prevalence	3·07(1·31–7·17)	2·51(0·72–8·78)	0·15(0·03–0·76)	2·52(0·97–6·81)	1·53(0·81–2·90)
Skin pinch						
	Immediate	ref	ref	ref	ref	ref
	1–2 s	1·17(0·94–1·44)	1·5(1·12–2·02)	1·26(0·83–1·93)	1·29(0·99–1·69)	1·17(0·94–1·44)
	> 2 s	1·47(1·09–1·98)	2·54(1·67–3·86)	2·28(1·16–4·46)	1·96(1·25–3·15)	1·47(1·09–1·98)
Capillary refill time						
	<=2 scs	ref	ref	ref	ref	ref
	>3 s	1·68(1·32–2·13)	1·08(0·67–1·76)	1·41(0·85–2·32)	1·39(0·95–2·07)	1·68(1·32–2·13)
	Indeterminate	1·83(1·12–2·99)	1·01(0·43–2·35)	na	1·27(0·51–1·75)	1·83(1·12–2·99)
Sunken eyes						
	No	ref	ref	ref	ref	ref
	Yes	1·24(1–1·55)	0·96(0·69–1·34)	1·04(0·74–1·46)	1·11(0·84–1·47)	1·24(1·00–1·55)
Jaundice						
	None	ref	ref	ref	ref	ref
	Moderate/severe	1·64(1·01–2·65)	1·72(0·96–3·1)	1·78(0·97–3·27)	1·71(0·98–2·98)	1·64(1·01–2·65)
Severe wasting^a^						
	Severe wasting no in high malaria prevalence	ref	ref	ref	ref	ref
	Severe wasting yes in high malaria prevalence	2·54(1·7–3·82)	2·41(1·27–4·58)	2·39(1·23–4·62)	2·46(1·44–4·26)	2·54(1·70–3·82)
	Severe wasting yes in low malaria prevalence	2·45(1·17–5·13)	3·27(1·09–9·79)	0·32(0·08–1·27)	2·29(0·94–5·79)	1·66(0·95–2·87)
Oedema of malnutrition^a^						
	Oedema none in high malaria prevalence	ref	ref	ref	ref	ref
	Oedema mild/moderate in high malaria prevalence	3·13(1·95–5·02)	2·92(1·44–5·92)	1·66(0·67–4·13)	2·78(1·55–5·10)	3·13(1·95–5·02)
	Oedema mild/moderate in Low malaria prevalence	1·94(0·96–3·90)	2·70(0·98–7·47)	0·45(0·11–1·91)	2·02(0·88–4·81)	1·88(1·08–3·25)
Malaria endemicity						
	High	ref	ref	ref	ref	ref
	Low	0·78(0·43–1·42)	1·12(0·49–2·53)	0·19(0·06–0·63)	0·76(0·38–1·58)	0·78(0·43–1·42)

Model 3 stratified by number of missing variables and weighted estimates across the missing patterns (assumes MNAR) compared to pooled model 3 data (assumes MAR)

^a^Variables with significant interactions with malaria endemicity

## Abbreviations

AUC: Area under the receiver operating curve
CIN: Clinical information network
CUSUM: Cumulative sum control charts
DHIS2: District Health Information system
EMR: Electronic medical records
KEMRI: Kenya Medical Research Institute
LRT: Likelihood ratio test
MAR: Missing at random
MNAR: Missing Not At Random
PAR: Pediatric admission record
PCV: Pneumococcal conjugate vaccine
REDCap: Research electronic data capture
RHIS: Routine health information system (RHIS
WAZ: Weight-for-age z-score
WHO: World Health Organization
